# Editorial: Methods in cardiovascular biologics and regenerative medicine

**DOI:** 10.3389/fcvm.2024.1477927

**Published:** 2024-09-13

**Authors:** Narasimman Gurusamy, Olaf Bergmann, Clotilde Castaldo, Ngan F. Huang, Ching-Ling Lien, Jun Jie Tan, Felix B. Engel

**Affiliations:** ^1^ Department of Pharmaceutical Sciences, Barry and Judy Silverman College of Pharmacy, Nova Southeastern University, Fort Lauderdale, FL, United States; ^2^ Department of Pharmacology and Toxicology, University Medical Center Goettingen, Goettingen, Germany; ^3^ Department of Cell and Molecular Biology, Karolinska Institute, Stockholm, Sweden; ^4^ DZHK (German Centre for Cardiovascular Research), Partner Site Lower Saxony, Lower Saxony, Germany; ^5^ Department of Public Health, School of Medicine and Surgery, University of Naples Federico II, Naples, Italy; ^6^ Department of Cardiothoracic Surgery, Stanford University, Palo Alto, CA, United States; ^7^ Center for Tissue Regeneration, Repair and Restoration, Veterans Affairs Palo Alto, Health Care System, Palo Alto, CA, United States; ^8^ Saban Research Institute, Children's Hospital Los Angeles, Los Angeles, CA, United States; ^9^ USM-ALPS Laboratory for Heart Research, Advanced Medical and Dental Institute, Universiti Sains Malaysia, Penang, Malaysia; ^10^ Department of Nephropathology, Institute of Pathology and Department of Cardiology, Experimental Renal and Cardiovascular Research, Friedrich-Alexander-Universität Erlangen-Nürnberg (FAU), Erlangen, Germany

**Keywords:** methods in cardiovascular biologics, regenerative medicine, tissue engineering, pericytes, cardiac remuscularization, 3D-bioprinting, vascular grafts, mitochondrial function

**Editorial on the Research Topic**
Methods in cardiovascular biologics and regenerative medicine

Cardiovascular diseases remain the leading cause of mortality globally, accounting for 20.5 million deaths in 2021, which is approximately one-third of all deaths annually (1). Given the complexity and substantial economic burden posed by cardiovascular risk factors and diseases (2, 3), it is imperative to develop innovative methodologies to improve treatment outcomes and deepen our understanding of cardiovascular pathophysiology. In response to this critical need, this Research Topic focuses on showcasing cutting-edge experimental techniques and methods in cardiovascular biologics and regenerative medicine. The primary objective of this collection is to highlight significant advances and novel approaches that are contributing to the growth of the field. These methodologies encompass a wide range of experimental strategies designed to address various aspects of cardiovascular health, from cellular and molecular mechanisms to tissue engineering and therapeutic interventions.

In the realm of cardiac remuscularization, large animal models are indispensable for translational research (4). The methodological review by Yu et al. offers a comprehensive evaluation of these models, discussing their advantages, limitations, and applications in preclinical studies. These models are crucial for testing the efficacy and safety of new regenerative therapies before they can be applied in human clinical trials. By improving our understanding of these models, this review helps to bridge the gap between laboratory research and clinical application.

The review by Alvino et al. on the isolation and long-term expansion of pericytes from human and animal tissues provides an overview of currently available methods for obtaining these critical cells and points out the challenges to create therapeutic pericyte products. Pericytes play a vital role in vascular biology. They are essential for maintaining vascular stability and function, making them a key target for regenerative therapies (5, 6). This method enhances our ability to study pericytes in detail, paving the way for developing new treatments for vascular diseases and might offer a solution for the vascularization of engineered tissues.

Tissue engineering and regenerative medicine have made significant strides with the development of decellularized vascular scaffolds enhanced with polyvinylidene fluoride and polycaprolactone reinforcement (7). The study by Klyshnikov et al. describes how this fused deposition modeling approach improves the mechanical properties and biocompatibility of vascular grafts, making them more suitable for clinical applications. This research aligns with the overarching goal of developing functional and durable vascular grafts for patients with cardiovascular diseases.

Advancements in 3D-bioprinting technology have the potential to reform the field of cardiovascular research (8, 9). Importantly, engineered 3D human heart models will reduce the need for animal experiments and might be more predictive. The article by Wolfe et al. on 3D-bioprinting of patient-derived cardiac tissue models highlights how this innovative technique can be used to create personalized cardiac tissues. These models are particularly useful for studying congenital heart disease, offering a platform for understanding disease mechanisms and testing therapeutic interventions in a patient-specific context. This approach aligns with the broader goal of personalized medicine, aiming to tailor treatments to individual patients’ needs.

Another approach to study cardiomyocytes in their environment and to reduce animal experiments and animal burden, is the use of living heart slices (10). A critical point hereby is to maintain heart tissue viability for studying heart physiology and disease mechanisms. The novel method developed by Ross et al. to extend the viability and functionality of living porcine heart slices from 1 to 6 days offers a valuable tool for ex vivo cardiac research. This technique enhances the utility of heart slices in research, facilitating more accurate studies of cardiac responses to various interventions and potentially leading to new therapeutic approaches.

Integrins play a critical role in cell adhesion and signaling, which are vital for vascularization, cardiac repair and regeneration as well as cardiac tissue engineering. Importantly, effective marketed treatments have successfully targeted integrins for cardiovascular diseases (11). A scientometric analysis by Lv et al. explores global research trends and emerging opportunities for integrin adhesion complexes in cardiac repair. This comprehensive overview identifies key areas of growth and potential in the field, guiding future studies and fostering international collaboration. Understanding these trends is essential for advancing cardiac repair strategies and improving patient outcomes.

Savchenko et al. examine the myocardial capacity of mitochondrial oxidative phosphorylation in response to prolonged electromagnetic stress, providing critical insights into cellular energy dynamics. Mitochondrial function is fundamental to cardiac health, and understanding how it is affected by external stressors can inform the development of therapies for myocardial dysfunction (12). This research contributes to the broader objective of improving cardiac function and resilience under various pathological conditions.

Besides vascular and muscular diseases, lymphedema is a significant clinical problem with limited treatment options (13). The development of a rat model of lymphedema and the implantation of a collagen-based medical device for therapeutic intervention by Nguyen et al. provides a novel platform for studying lymphatic disorders. This model enables the evaluation of new therapies in a controlled setting, contributing to the broader goal of developing effective treatments for lymphatic diseases and improving patient quality of life.

This collection of articles underscores the innovative methods being developed and emphasizes the collaborative effort required to tackle cardiovascular challenges. The integration of diverse techniques—from pericyte isolation and the use of living heart slices to advanced tissue engineering including 3D-bioprinting of cardiac tissues to the use of large animal models—demonstrates a multifaceted approach essential for driving progress in cardiovascular research. These methodologies, poised to significantly impact future therapeutic strategies, include personalized cardiac tissue models for patient-specific treatments and advanced vascular grafts for clinical applications. As we continue to explore and refine these methodologies, the contributions in this Research Topic will undoubtedly pave the way for future advances in cardiovascular biologics and regenerative medicine, ultimately improving patient outcomes and reducing the global burden of cardiovascular disease.
